# Supplementary material for: Towards maximized volumetric capacity via pore-coordinated design for large-volume-change lithium-ion battery anodes
Source: Nat Commun. 2019 Jan 29;10:475. doi: 10.1038/s41467-018-08233-3 (PMC6351620; doi:10.1038/s41467-018-08233-3)
Supplement: Supplementary file 1 — Supplementary Information [file 41467_2018_8233_MOESM1_ESM.docx]

**Supplementary Information**

**Towards Maximized Volumetric Capacity via Pore-coordinated Design for Large-volume-change Lithium-ion Battery Anodes**

Ma et al.

**
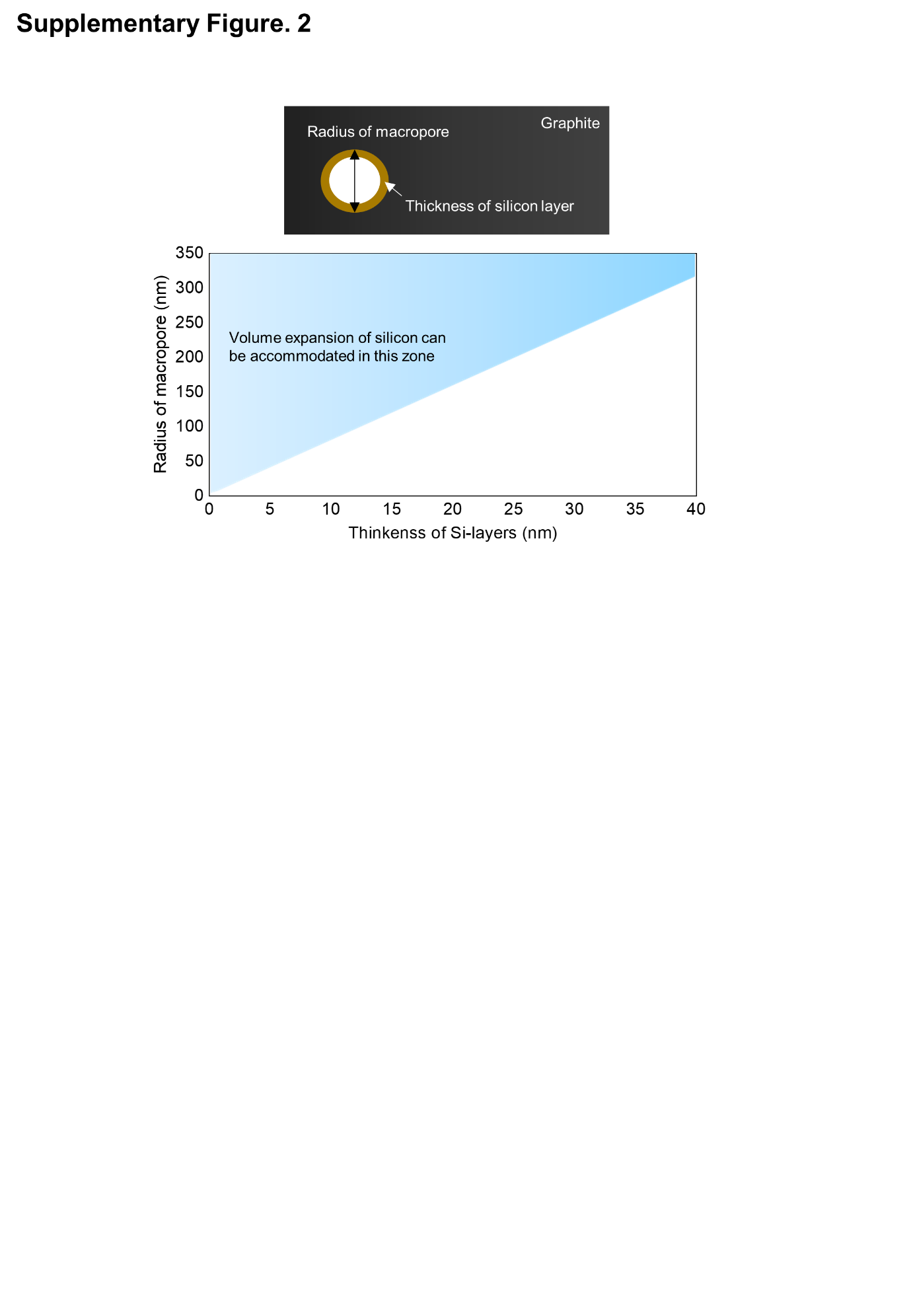
**

**Supplementary Figure 1**. The graph suggests the minimum pore-size required to accommodate volume expansion depending on a thickness of Si-layers represented in a schematic illustration (Detailed description is given in Supplementary Note 2).


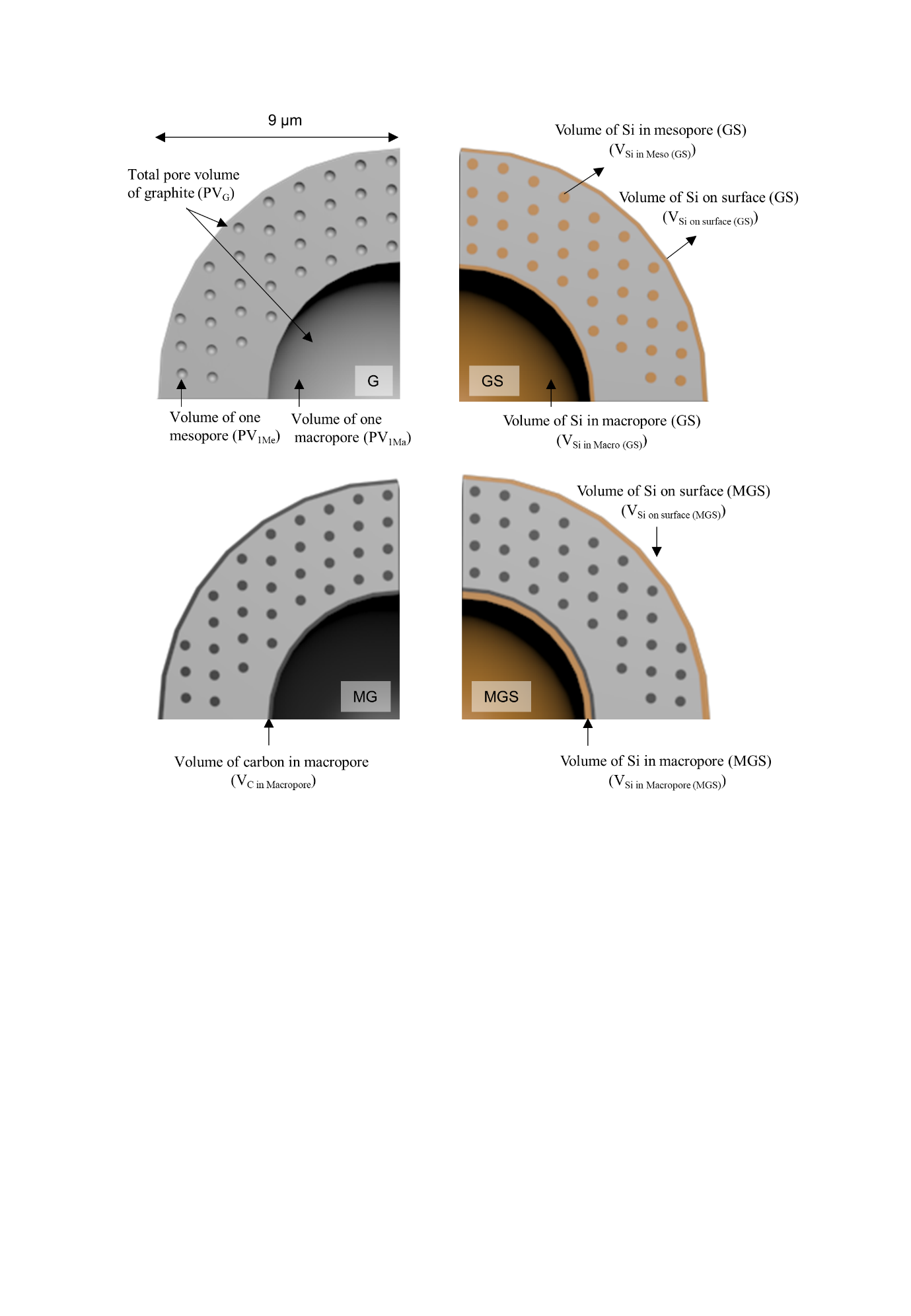


**Supplementary Figure 2**. The terms (Supplementary Note 3) are represented in the schematic illustration of G, GS, MG, and MGS.


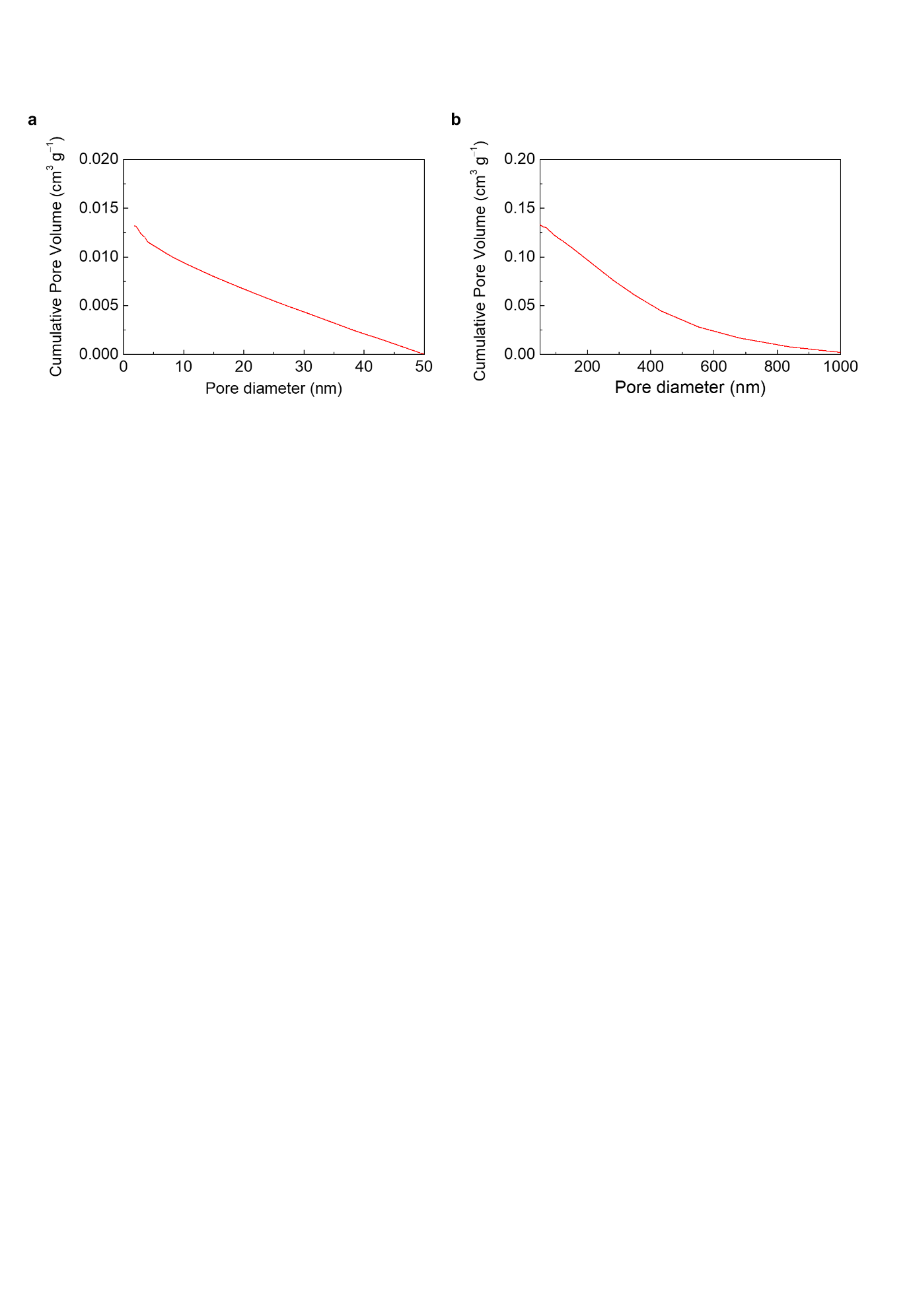


**Supplementary Figure 3**. Graph of the cumulative pore volume of G as a function of pore diameter by (a) BJH method and (b) mercury intrusion porosimetry.


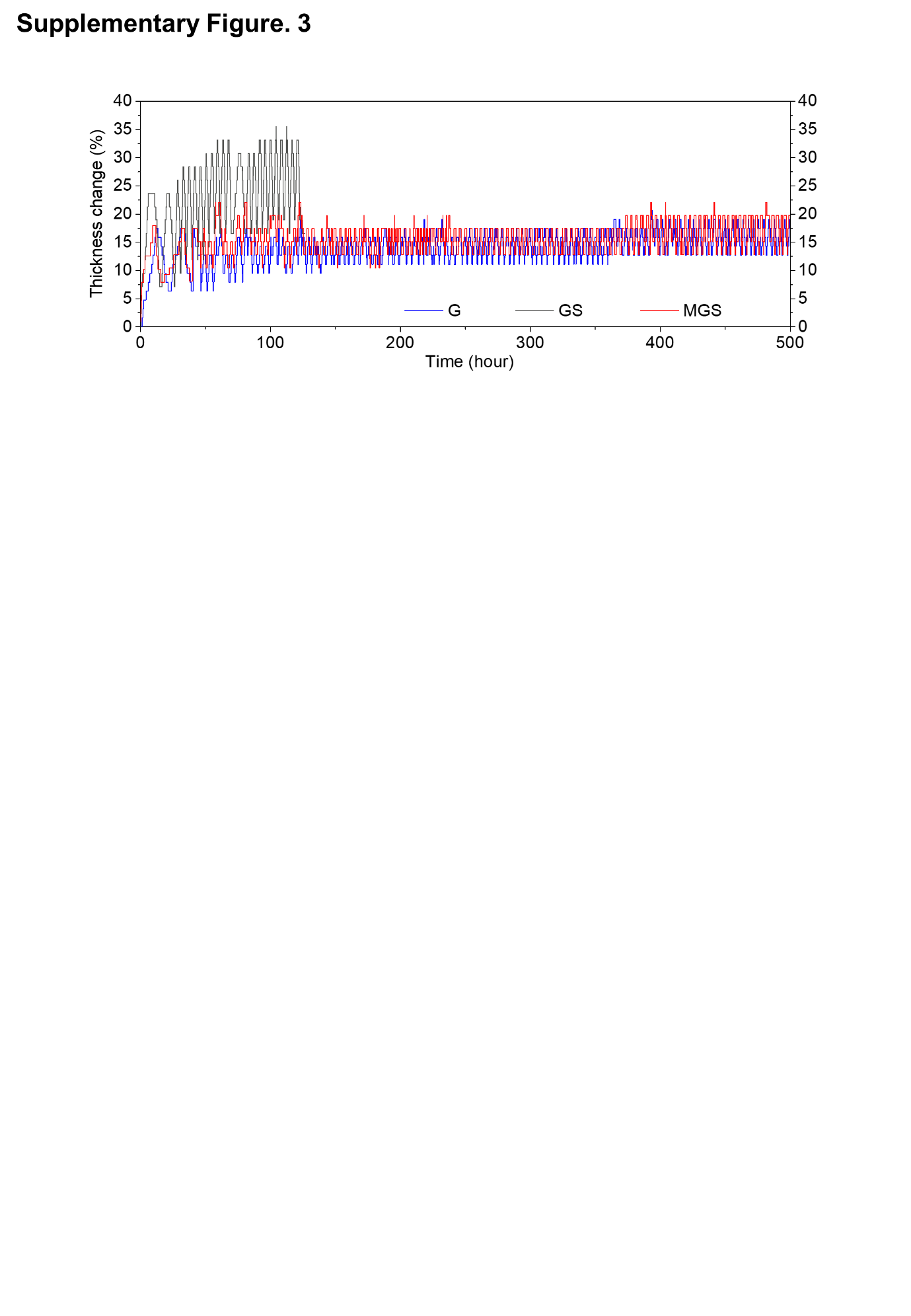


**Supplementary Figure 4**. Graph of the ratio of the thickness change of G, GS, and MGS as a function of charge-discharge time. (500 hours corresponds to about 100 cycles)


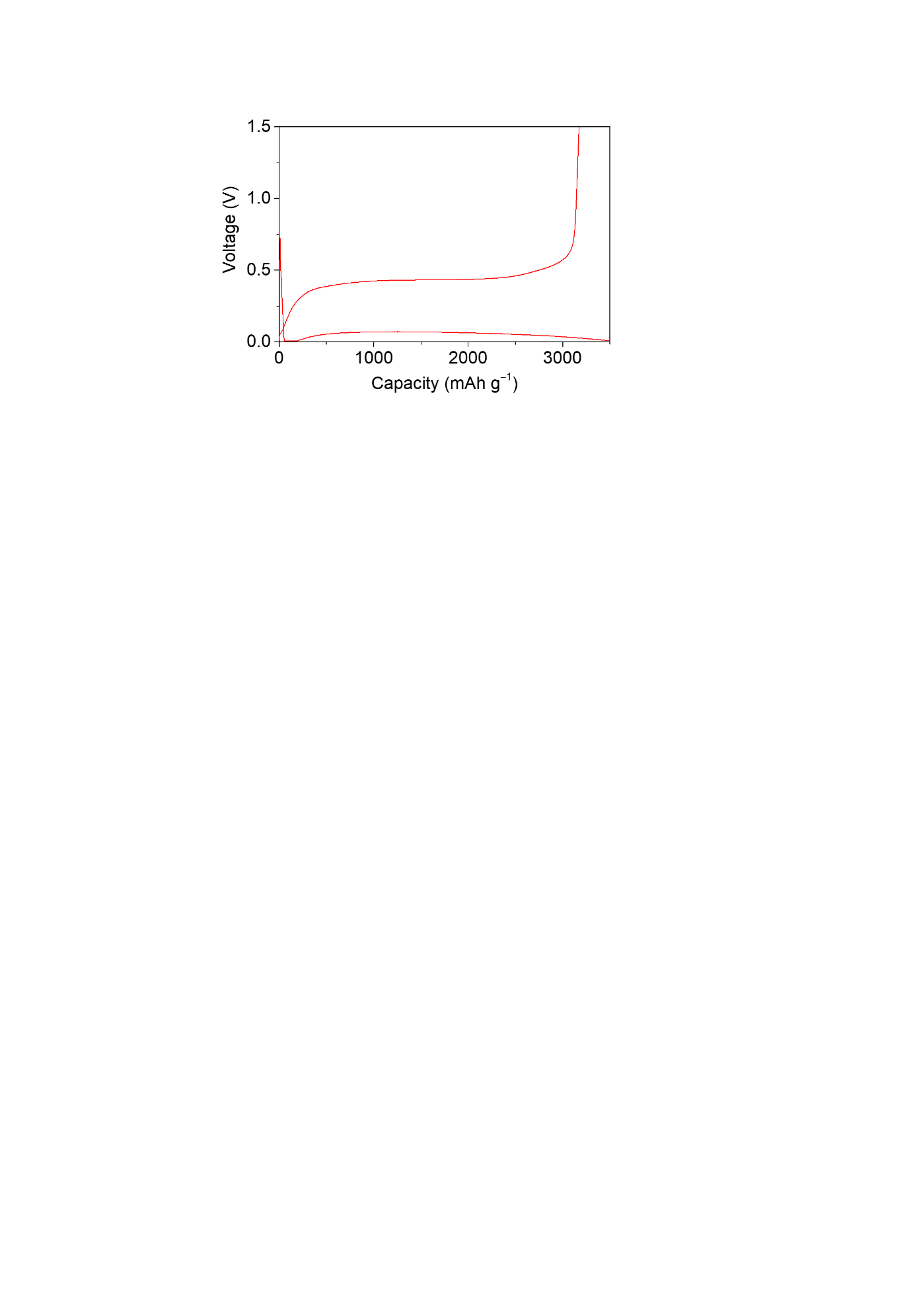


**Supplementary Figure 5**. Voltage profile of the nano-Si powders synthesized by thermal decomposition of monosilane for the first cycle at 0.1 C in a coin-type half-cell at 24 ^o^C.


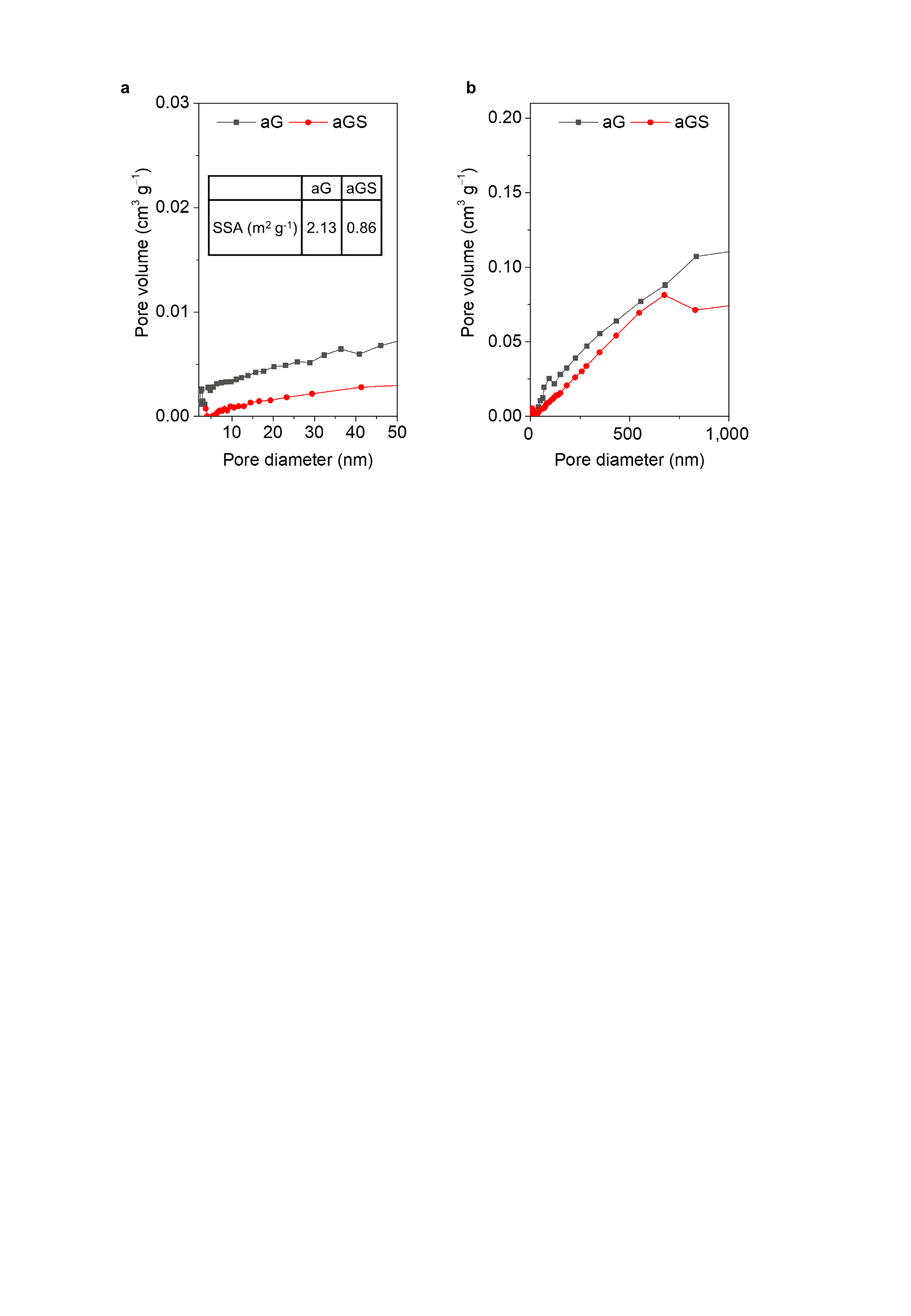


**Supplementary Figure 6**. Mesoporous size distribution by BJH method of aG and aGS. The inset shows their specific surface area (SSA).


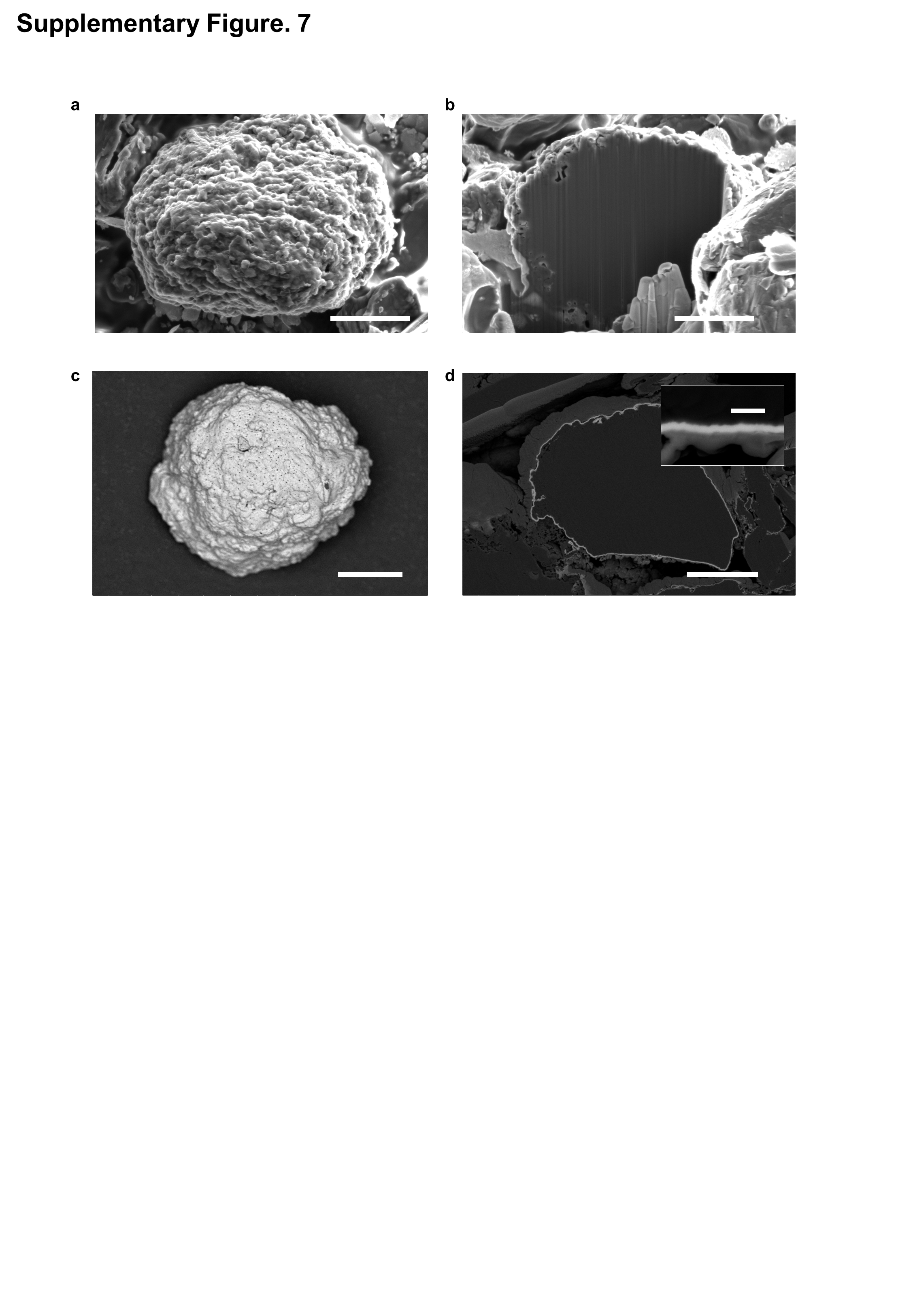


**Supplementary Figure 7**. Top view of SEM images of (a) aG and (c) aGS and cross-sectional images of (b) aG and (d) aGS with an inset showing high magnified surface of aGS after ion beam milling. Scale bars, 5 μm (a−d) and 300 nm (inset in d).


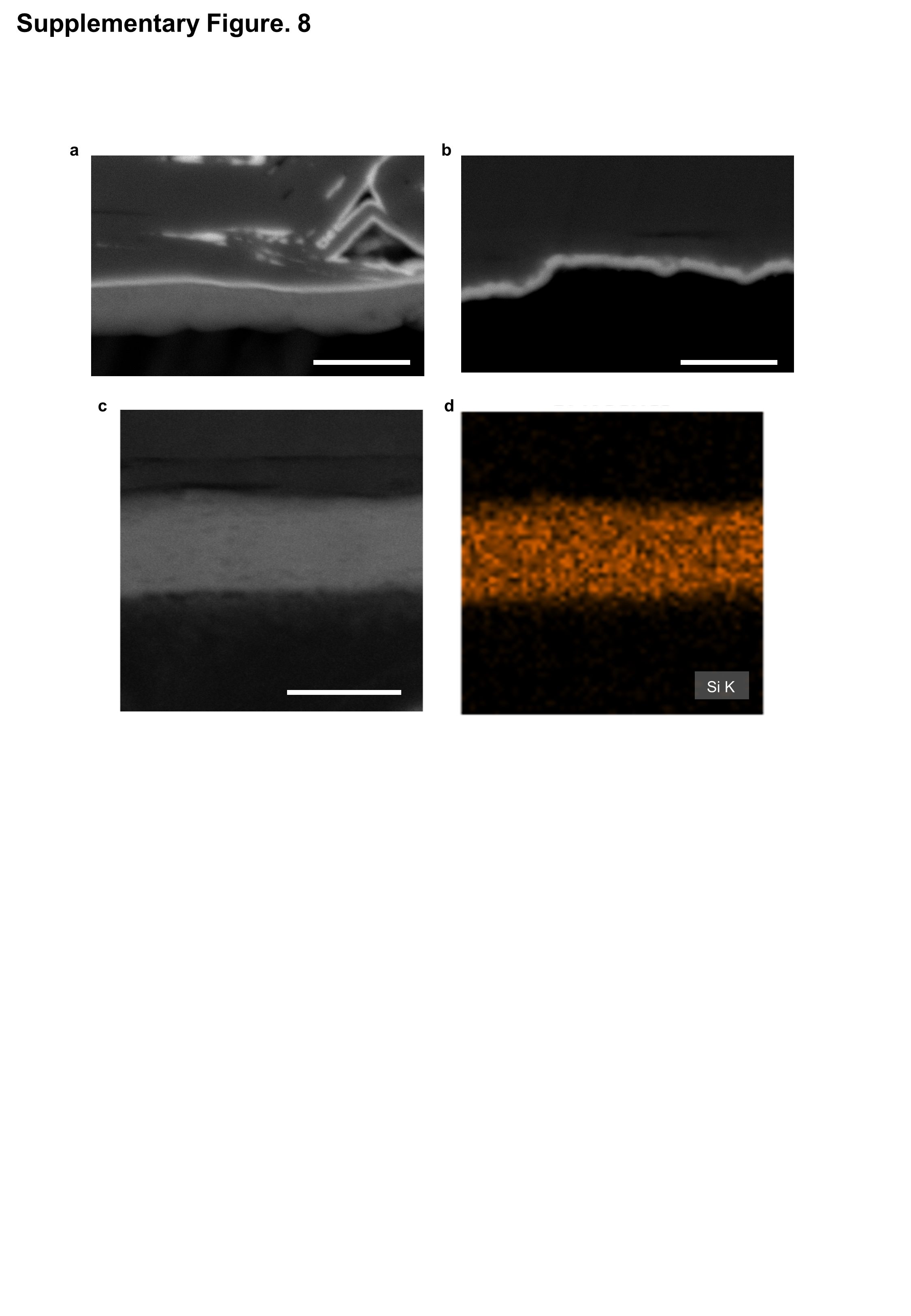


**Supplementary Figure 8**. SEM images exhibit the Si-layers of (a) GS and (b) MGS with the thicknesses ~20 nm and 30 nm, respectively. (c) Cross-sectional STEM images and (d) elemental mapping by energy-dispersive spectroscopy for Si-blocking in GS before cycling (the thickness of Si-blocking is about 40 nm). Scale bars, 300 nm (a,b) and 50 nm (c).


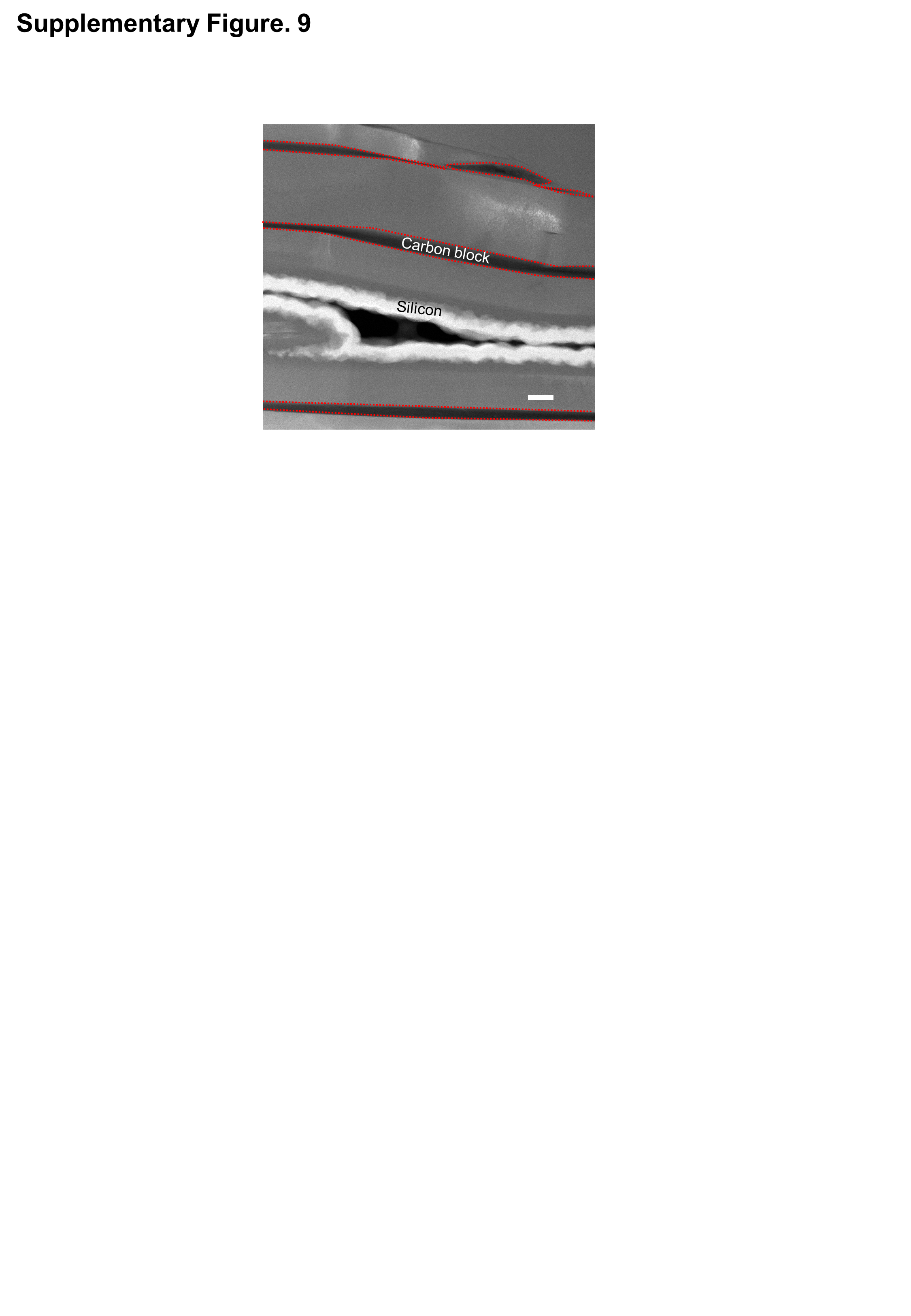


**Supplementary Figure 9**. STEM image of MGS represents that carbon-blocking in mesopores is in stark contrast to the Si-layers in the macropore. Scale bars, 50 nm.


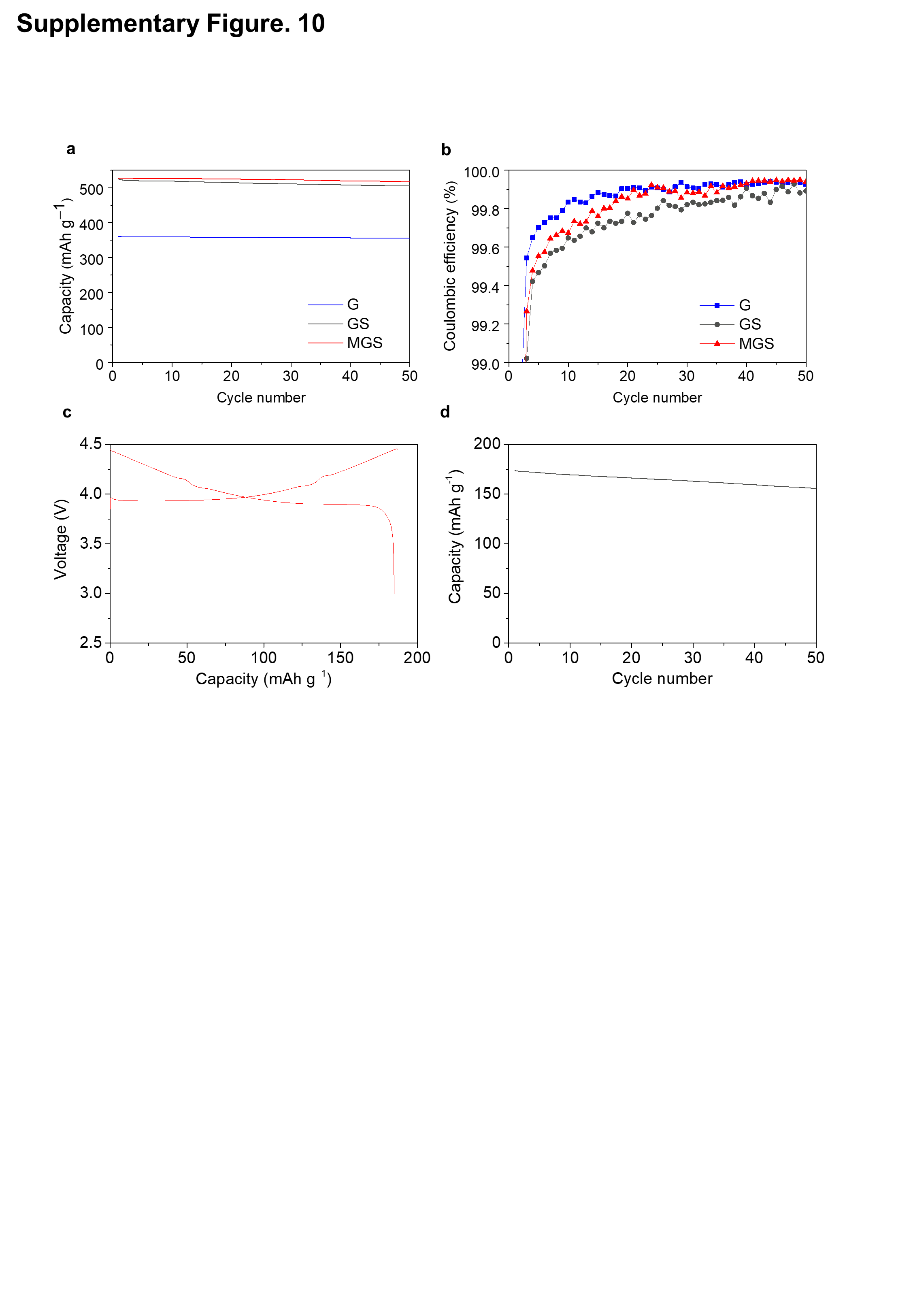


**Supplementary Figure 10**. (a) Reversible discharge capacity and (b) CE of G, GS and MGS during 50 cycles at 0.5 C in a coin-type half-cell (discharge capacity and CE for the first cycle was excluded). (c) Voltage profile of LiCoO_2_ in a coin type half-cell and (d) reversible discharge capacity at 0.5 C during 50 cycles in the potential window between 3.0 V and 4.45 V.


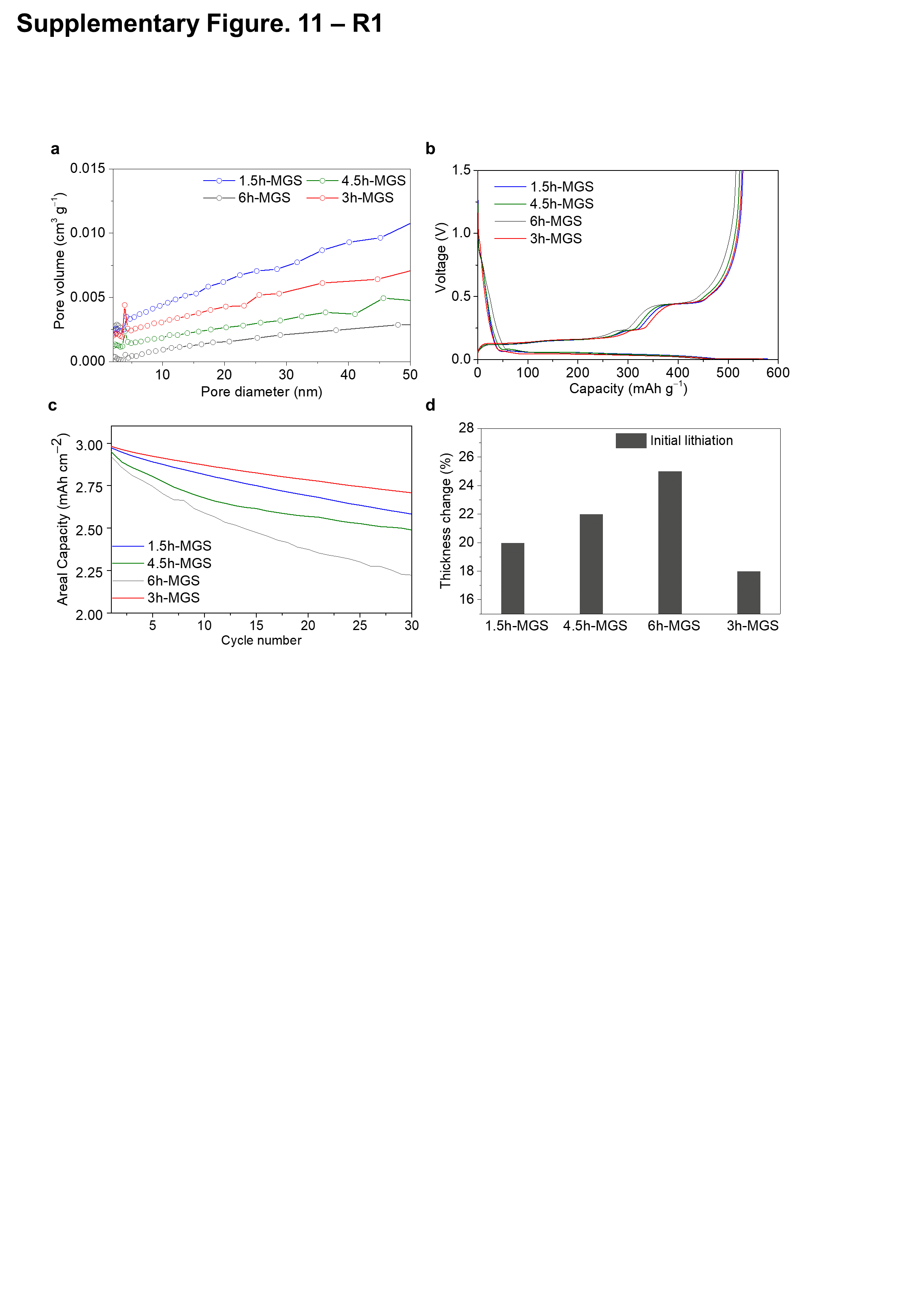


**Supplementary Figure 11**. Comparison of several-typed MGSs synthesized with different ethylene flow time for 1.5 h (1.5h-MGS), 3 h (3h-MGS), 4.5 h (4.5h-MGS) and 6 h (6h-MGS). (a) Mesopore size distribution determined via BJH method. (b) Voltage profiles for the first cycles at 0.1 C in half-cell and (c) cycling performances at 1 C in full-cell. (d) Thickness changes after the first lithiation of each sample.


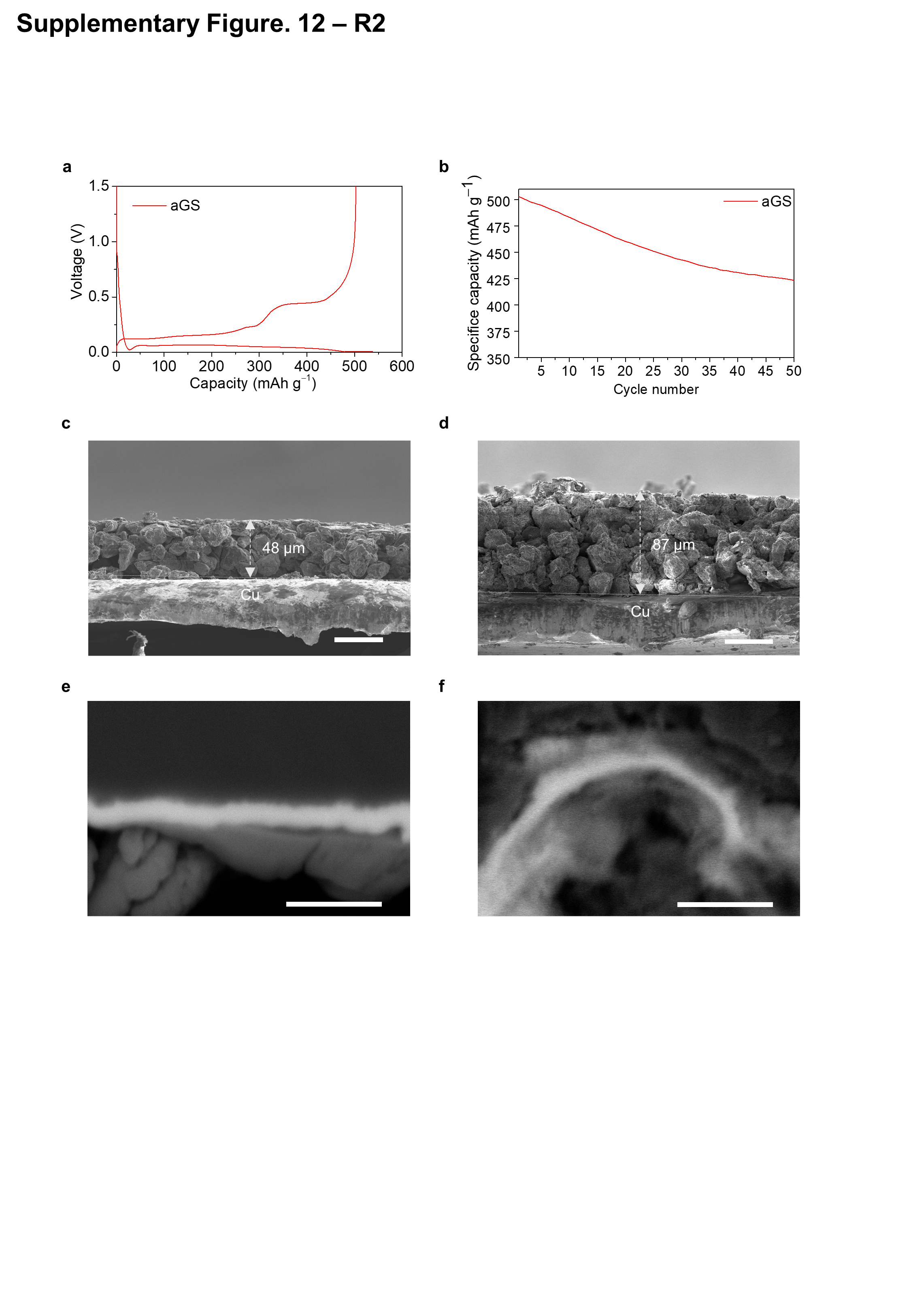


**Supplementary Figure 12**. Electrochemical characterization and analysis of aGS (a) Voltage profile for the first cycle and (b) cycling performance of aGS at 0.5 C in half-cell. Cross-sectional views of aGS electrode and surface SEM images (c, e) before cycling and (d, f) after 100 cycles, respectively. Scale bars, 50 μm (c,d) and 300 nm (e,f).


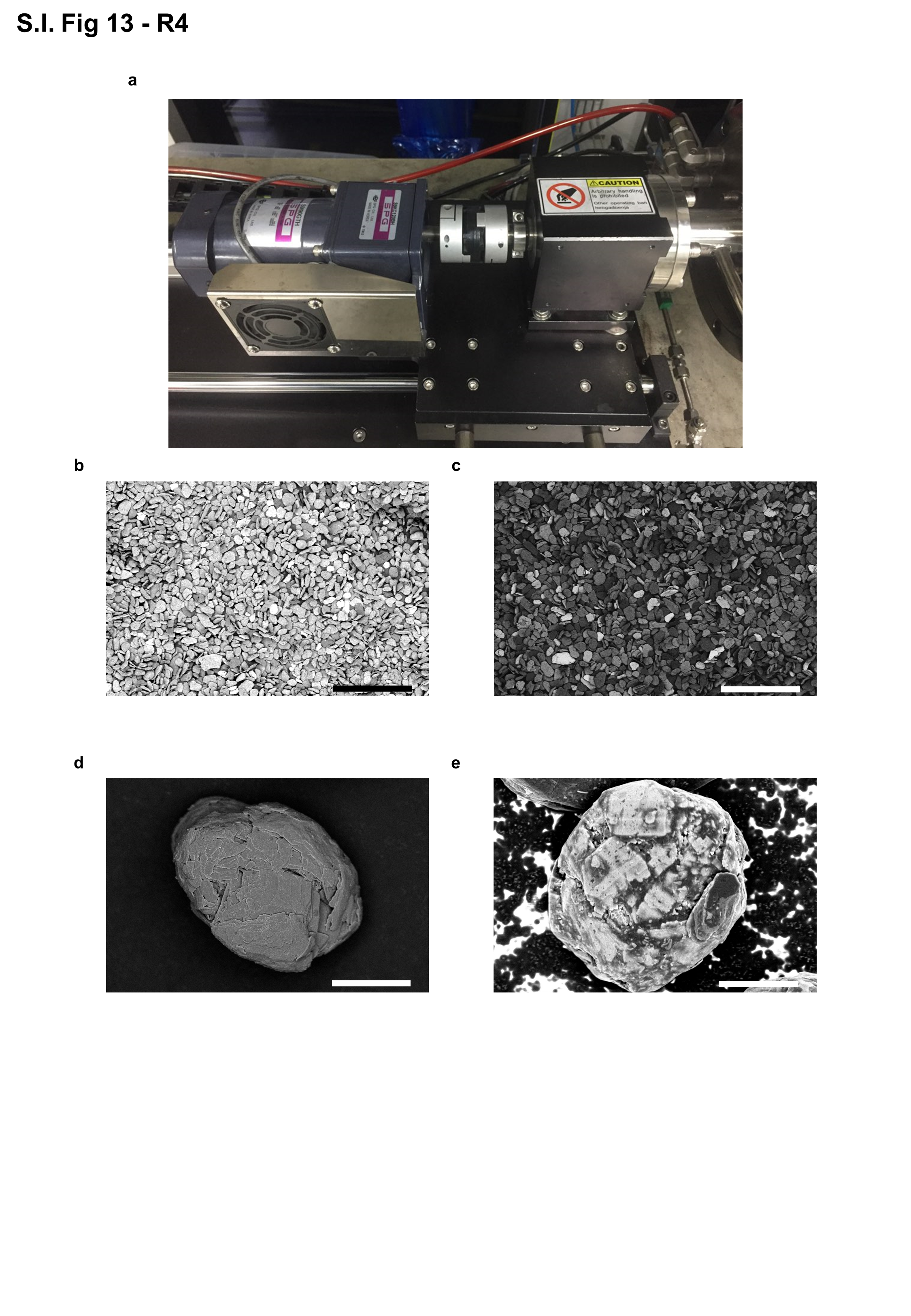


**Supplementary Figure 13**. (a) The rotational driving system of customized rotatable CVD furnace. SEM images of MGS synthesized through (b, d) the rotation mode and (c, e) the non-rotation, which show superior uniformity of deposition and high nonuniformity of silicon deposition, respectively. Scale bars, 100 μm (b,c) and 5 μm (d,e).


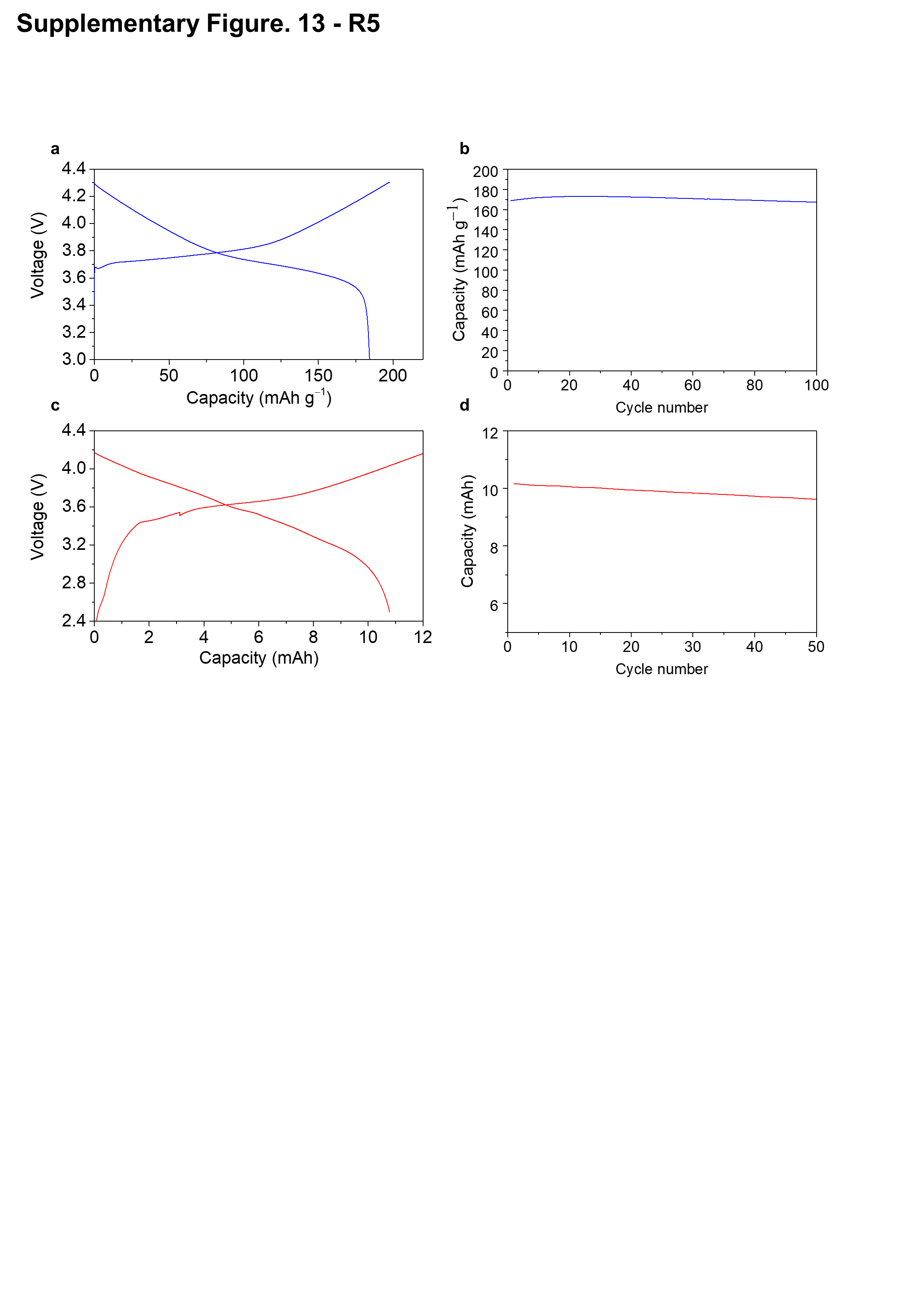


**Supplementary Figure 14**. Voltage profiles and cycling performances of LiNi_0.6_Co_0.2_Mn_0.2_O_2_, which is widely used in automotive applications, (a, b) half-cell and (c, d) full-cell paired with the MGS, respectively.


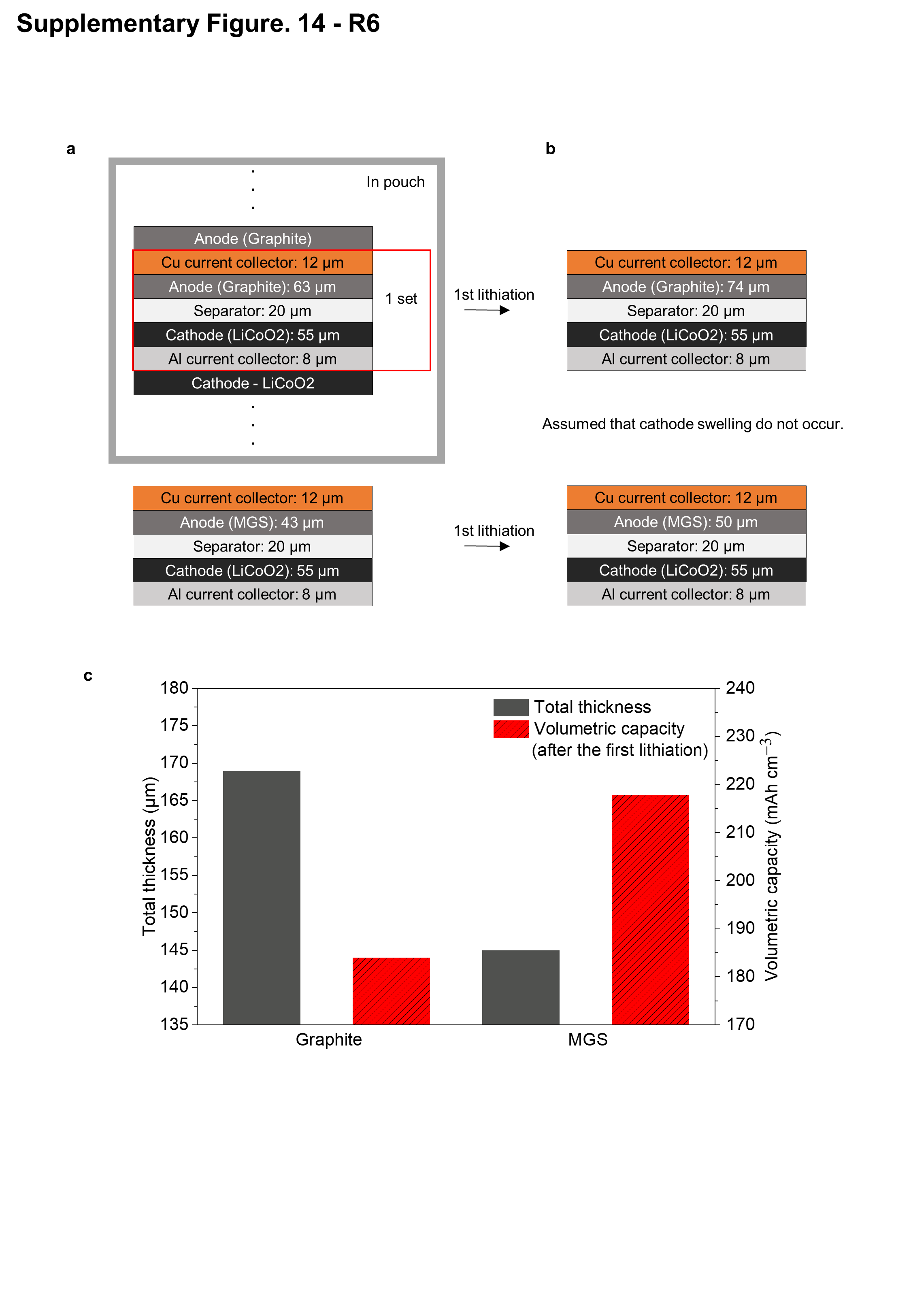


**Supplementary Figure 15**. Schematic illustration including thickness information of pouch full-cell at the industrial level comprising graphite and MGS (a) before cycling and (b) after the first lithiation. (c) Total thickness and volumetric capacity of MGS and graphite full-cell, considering the thickness of both electrodes, current collectors and membrane.


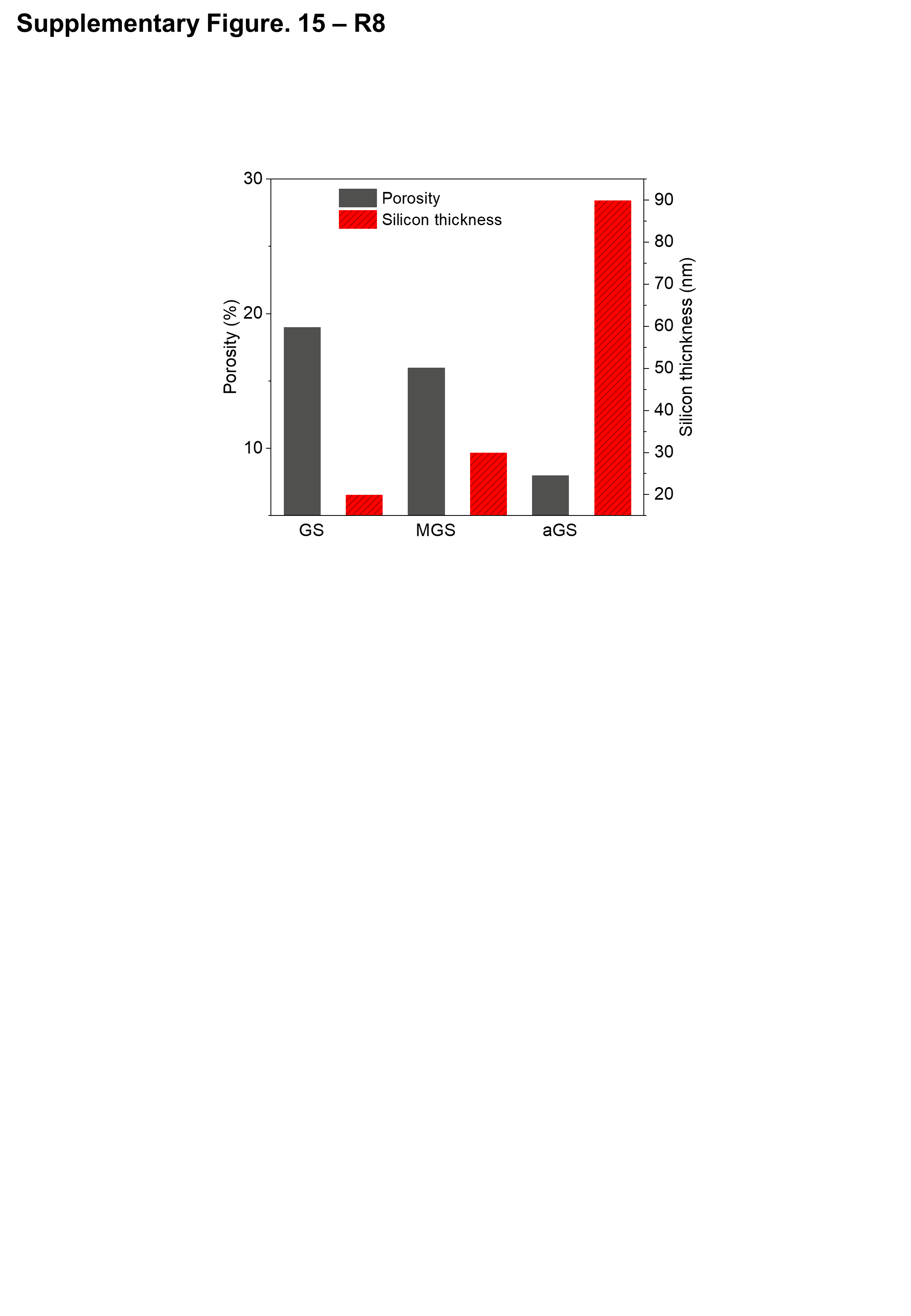


**Supplementary Figure 16**. Porosity and silicon thickness of GS, MGS and aGS, respectively.


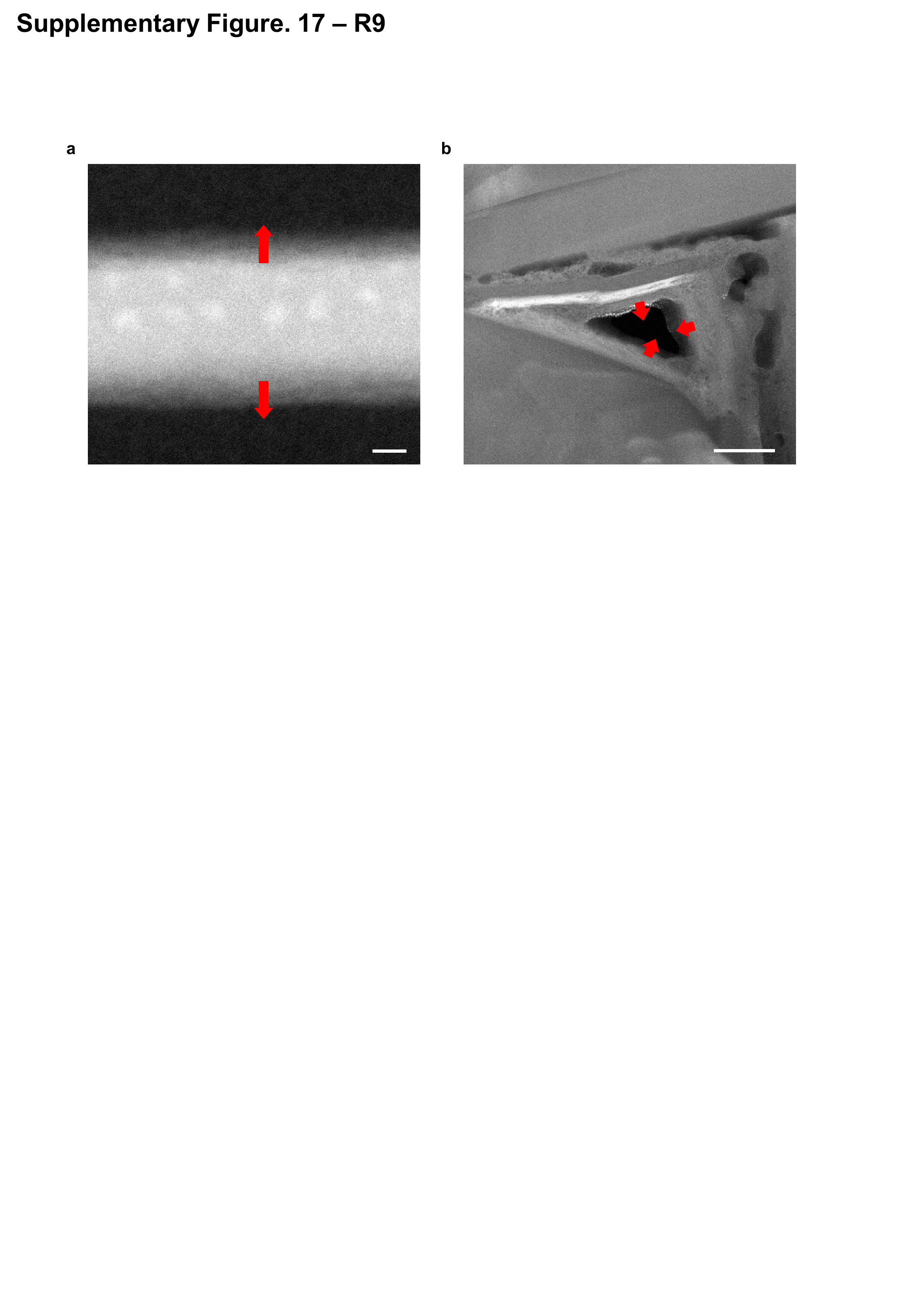


**Supplementary Figure 17**. TEM images after the initial expansion of silicon in (a) mesopore and (b) macropore. Scale bars, 5 nm (a) and 100 nm (b).


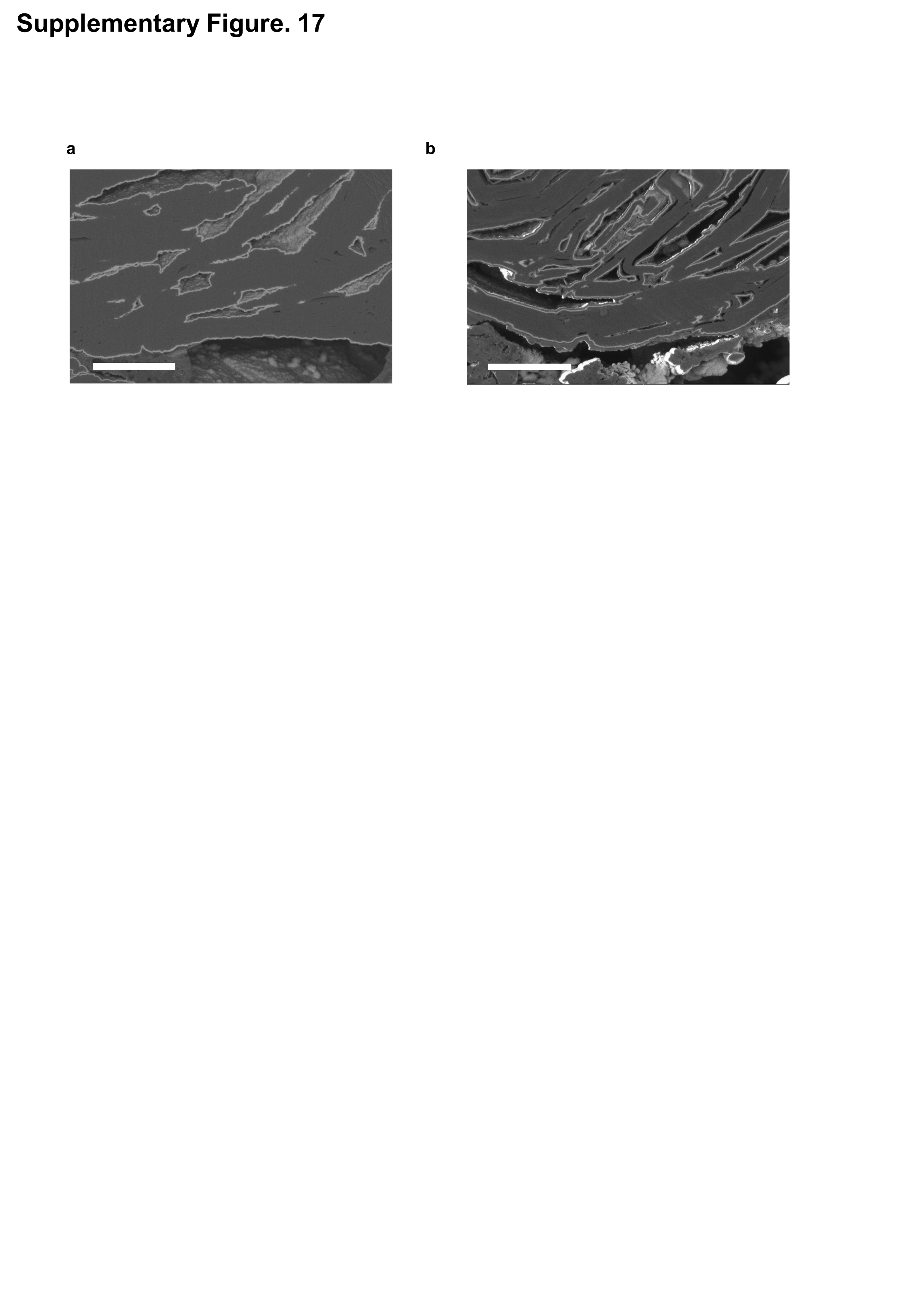


**Supplementary Figure 18**. Demagnified SEM images of (a) Fig. 4g and (b) i. Scale bars, 2 μm (a,b).

**Supplementary Table 1**. Details of each term in Supplementary Note. 3

| symbol | name | Equation | | Value | unit |
| --- | --- | --- | --- | --- | --- |
| V_G_ | Volume of graphite | $\pi\frac{4}{3}{9000}^{3}$ | 3.05 × 10^12^ | | nm^3^ |
| PV_G_ | Total pore volume of graphite | $0.23V_{G}$ | 7.02 × 10^11^ | | nm^3^ |
| TV_G_ | True volume of graphite | $0.77V_{G}$ | 2.35 × 10^12^ | | nm^3^ |
| V_Si_ | Total volume of Si in graphite | $\frac{{6.3TV}_{G}}{93.7}$ | 1.58 × 10^11^ | | nm^3^ |
| PV_Me_ / PV_Ma_ | Total volume of mesopores/macropores | ${PV}_{G}\frac{1}{11}$ */* ${PV}_{G}\frac{10}{11}$ | 6.38 × 10^10^ / 6.38 × 10^11^ | | nm^3^ |
| PV_1Me_ / PV_1Ma_ | Volume of one mesopores/macropores | $\pi\frac{4}{3}{20}^{3}$ */* $\pi\frac{4}{3}{500}^{3}$ | 3.35 × 10^4^ / 5.24 × 10^8^ | | nm^3^ |
| N_Me_ / N_Ma_ | Number of mesopores/macropores | $\frac{{PV}_{Me}}{{PV}_{1Me}} / \frac{{PV}_{Ma}}{{PV}_{1Ma}}$ | 1.90 × 10^6^ / 1.22 × 10^3^ | |  |
| V_C in Macropores_ | Volume of carbon in macropores (MG and MGS) | $N_{Ma}[\{\pi\frac{4}{3}\left\{ {500}^{3}-\left( 500-20 \right)^{3} \right\}]$ | 7.36 × 10^10^ | | nm^3^ |
| V_Si in Meso (GS)_ | Volume of Si in mesopores (GS) | ${PV}_{Me}$ | 6.38 × 10^10^ | | nm^3^ |
| V_Si in Macro (GS)_ | Volume of Si in macropores (GS) | $V_{C in Macropores}$ | 7.36 × 10^10^ | | nm^3^ |
| V_Si on surface (GS)_ | Volume of Si on surface (GS) | $\pi\frac{4}{3}{9020}^{3}- V_{G}$ | 2.04 × 10^10^ | | nm^3^ |
| V_Si in Macro (MGS)_ | Volume of Si in macropores (MGS) | $N_{Ma}[\pi\frac{4}{3}\{{\left( 500-20 \right)^{3}-(500-20-36.8)\}}^{3}]$ | 1.20 × 10^11^ | | nm^3^ |
| V_Si on surface (MGS)_ | Volume of Si on surface (MGS) | $\pi\frac{4}{3}{(9000+20+36.8)}^{3}- V_{G}$ | 3.78 × 10^10^ | | nm^3^ |
| P_theo of MG_ | Theoretical porosity (MG) | $23-23\frac{{{PV}_{Me}+ V}_{C in Macropores}}{{PV}_{G}}$ | 18.5 | | % |
| P_theo of GS_ | Theoretical porosity (GS) | *P_theo of MG_* | 18.5 | | % |
| P_theo of MGS_ | Theoretical porosity (MGS) | $23-23\frac{{{PV}_{Me}+ V}_{Si in Macro \left( MGS \right)}{+ V}_{C in Macropores}}{{PV}_{G}}$ | 14.6 | | % |

**Supplementary Table 2**. Physical properties of materials in this study.

|  | | G | MG | GS | MGS |
| --- | --- | --- | --- | --- | --- |
| PSD (μm) | D10 | 12.71 | 14.10 | 13.41 | 14.95 |
|  | D50 | 18.3 | 19.36 | 18.88 | 19.81 |
|  | D90 | 27 | 27.70 | 27.19 | 28.01 |
| SSA (m^2^ g^−1^) | | 5.28 | 3.13 | 3.19 | 2.1 |
| Pore volume (cm^3^g^−1^) | | 0.022 | 0.012 | 0.014 | 0.0083 |
| Porosity (%) | | 23 | 20 | 19 | 16 |
| TD (g cm^−3^) | | 1.02 | 1.08 | 1.07 | 1.13 |

| **Volumetric capacity** | | | **Volumetric energy density** | | | | | |
| --- | --- | --- | --- | --- | --- | --- | --- | --- |
| $\frac{\text{(Areal cell capacity) }}{\text{(thickness of anode)}}$ | | | $\frac{\text{(Areal cell capacity) × (Average voltage)}}{\text{(thickness of anode)}}$ | | | | | |
| **Half-cell electrode information** | | | | | | | | |
|  | LCO | G | | | | GS | | MGS |
| Mass loading level (mg cm^–2^) | 20 | 10.1 | | | | 6.9 | | 6.9 |
| Electrode density (g cm^–3^) | 3.6 | 1.6 | | | | 1.6 | | 1.6 |
| Electrode thickness (μm) (excepting current collector) | 55 | 63 | | | | 43 | | 43 |
| Specific capacity (mAh g^-1^) | 184.8 | 360 | | | | 525 | | 527 |
| Initial coulombic efficiency (%) | 98.6 | 92.0 | | | | 92.2 | | 93.0 |
| **Full-cell information** | | | | | | |  |  |
| 1^st^ cycle | | | | | | |  |  |
|  | G | | | GS | MGS | |  |  |
| Areal cell capacity (mAh cm^–2^) | 3.11 | | | 3.12 | 3.16 | |  |  |
| Coulombic efficiency (%) | 90.0 | | | 90.3 | 91.3 | |  |  |
| Average voltage (V) | 3.85 | | | 3.78 | 3.74 | |  |  |
| Electrode thickness  at fully-charged state (μm) | 74 | | | 53 | 50 | |  |  |
| Rate of electrode swelling (%) | 16 | | | 23 | 17 | |  |  |
| Volumetric capacity (mAh g^-1^) | 420.2 | | | 588.6 | 632.0 | |  |  |
| Volumetric energy density (Wh L^-1^) | 1619.2 | | | 2225.2 | 2363.6 | |  |  |
| 100^th^ cycle | | | | | | |  |  |
|  | G | | | GS | MGS | |  |  |
| Areal cell capacity (mAh cm^–2^) | 2.71 | | | 2.13 | 2.51 | |  |  |
| Average voltage (V) | 3.80 | | | 3.74 | 3.70 | |  |  |
| Electrode thickness  at fully-charged state (μm) | 74 | | | 58 | 51 | |  |  |
| Rate of electrode swelling (%) | 17 | | | 35 | 19 | |  |  |
| Volumetric capacity (mAh g^-1^) | 361.4 | | | 367.5 | 493.9 | |  |  |
| Volumetric energy density (Wh L^-1^) | 1376.3 | | | 1374.5 | 1825.7 | |  |  |

**Supplementary Table 3**. Volumetric capacity and energy density calculation including electrode information.

**Supplementary Note 1**. **Definition of volumetric capacity**

Loading level (LL) is defined as follows, using areal capacity (Q_a_), gravimetric capacity (Q_g_) and the active material ratio (R_am_). And original electrode thickness (T_oe_) is described by LL and electrode density (D_e_).

$$LL=\frac{Q_{a}}{Q_{g}R_{am}} (1)$$

$$T_{oe}=\frac{LL}{D_{e}}= \frac{Q_{a}}{Q_{g}R_{am}D_{e}} (2)$$

When we assume that the anode and cathode are in contact with the same area, volumetric capacity (Q_v_) is defined as follows using N/P ratio (R_np_) and swelling ratio (R_s_) and illustrated in Fig. 1a.

$$Q_{v}=\frac{Q_{a}}{T_{oe}R_{np}R_{s}}=\frac{Q_{g}R_{am}D_{e}}{R_{np}R_{s}} (3)$$

**Supplementary Note 2**. **Pore design of spherical graphite for accommodating volume expansion of Si**

Securing the external space to accommodate volume expansion of Si is an important issue of Si-based anode. To calculate macropore size containing enough space for Si-layers, we hypothesized that pore was completely sphere and Si expands volumetrically by up to 300% on full lithiated states. The relationship between the thickness of Si-layers (T_si_) and the radius of macropore (R_ma_) can be described in equation (5) via equation (4) and Supplementary Fig. 1.

$$3\frac{4}{3}\pi{\{{R_{ma}}^{3}-(R_{ma}-T_{si})}^{3}\}=\pi\frac{4}{3}{R_{ma}}^{3} (4)$$

$$R_{ma}=\frac{T_{si}}{1-\left( \frac{2}{3} \right)^{\frac{1}{3}}} (5)$$

For instance, when Si-layers with 20 nm or 36.8 nm is coated on pore of graphite, macropore size (diameter) is required over 316 nm or 582 nm to accommodate volume expansion of Si, respectively.

**Supplementary Note 3**. **Calculating numerical values for finite element model**

Based on the results of the BJH method and mercury-porosimetry (Supplementary Fig. 3), we set that volume ratio of the mesopores and macropores is 1:10 in G. From equation (6), we also calculated this real porosity (P_real_) which was 23% using bulk density (D_b_) and true density (D_T_).

$$P_{real}=\frac{{D_{T}-D}_{b}}{D_{T}} (6)$$

In the model, we assume that the spherical graphite is a perfect sphere with a diameter of 18 μm and density of graphite and Si is same. Also, the radius of mesopore and macropore are fixed at 20 nm and 500 nm, respectively. The thickness of Si-layers in GS and carbon-layers (carbon-blocking) in MGS is 20 nm based on the TEM images (Fig. 2e, and Supplementary Fig. 8, 9). From equation (7) - (10), we obtain the volume and number of mesopores and macropores, respectively. V_G_, PV_Me_, PV_Ma_, N_Me_, N_Ma_, represent the total volume of graphite, mesopores, macropores, and number of mesopores, macropores, respectively.

$${{PV}_{me}= {PV}_{G}\frac{1}{11} \left( 7 \right) {PV}_{ma}=PV}_{G}\frac{10}{11} (8)$$

$$N_{Me}=\frac{{PV}_{Me}}{{PV}_{1Me}} \left( 9 \right) N_{Ma}= \frac{{PV}_{Ma}}{{PV}_{1Ma}} (10)$$

The theoretical porosity of MG (P_theo of MG_), calculated using the volume of carbon in mesopores (V_C in Mesopores_) and macropores (V_C in Macropores_), is 18.5% from equation (11).

$$P_{theo of MG}=23-23\frac{{{PV}_{Me}+ V}_{C in Macropores}}{{PV}_{G}} \left( 11 \right)$$

To understand how much Si is coated on graphite in GS, we obtained the gravimetric capacity of G (360 mAh g^-1^), GS (525 mAh g^-1^) and pure Si (3000 mAh g^-1^), which was synthesized via thermal decomposition of monosilane gas (Supplementary Fig. 5). Then, the equation (12) is used to calculate the weight percent of Si in GS (6.3 wt%). When the same amount of Si is coated on G and MG models, the thickness of Si-layers was set at 20 nm and 36.8 nm, respectively.

$\left\{ 360\times\left( 1-x \right) \right\}+ \left( 3000 \times x \right) \approx525, x = 0.063$(12)

Using equation (13) - (18), we calculated Si distribution in GS. Total weight and volume fraction of Si (6.3 wt%, 100 vol%) in GS are distributed in the mesopores (2.6 wt%, 40.5 vol%), macropores (2.9 wt%, 46.6 vol%), and on the surface (0.8 wt%, 12.9 vol%). And the theoretical porosity of GS (18.5%) is same as that of MG. TV_G_, V_si_, V_Si in Meso (GS)_, V_Si in Macro (GS)_, V_Si on surface (GS)_ and P_theo of GS_ represent the true volume of graphite, total volume of Si in graphite, mesopores, macropores, the volume of Si on the surface and theoretical porosity of GS, respectively.

$${TV}_{G}= 0.77V_{G} (13)$$

$$V_{Si}= \frac{{6.3TV}_{G}}{93.7} (14)$$

$$V_{Si in Meso(GS)}= {PV}_{G}\frac{1}{11} (15)$$

$$V_{Si in Macro\left( GS \right)}= V_{C in Macropores} (16)$$

$$V_{Si in surface\left( GS \right)}= \pi\frac{4}{3}{9020}^{\frac{1}{3}}- V_{G} \left( 17 \right)$$

$$P_{theo of GS}=P_{theo of MG} (18)$$

Following equation (19) - (21), we also calculated Si distribution in MGS. Total weight and volume fraction of Si (6.3 wt%, 100 vol%) in MGS are distributed in the macropores (4.8 wt%, 76 vol%) and on the surface (1.5 wt%, 24 vol%). V_Si in Macro (MGS),_ V_Si on surface (MGS),_ P_theo of MGS_ represent the volume of Si in macropores, the volume of Si on the surface and theoretical porosity of MGS. These results show that most of the Si (72.8%) in GS mesopores is in MGS macropores. And the theoretical porosity of MGS is 14.6%.

$${V_{Si in Macro \left( MGS \right)}=N}_{Ma}[\pi\frac{4}{3}\left\{ {\left( 500-20 \right)^{3}-(500-20-36.8)\}}^{3} \right] (19)$$

$V_{Si on surface\left( MGS \right)}=\pi\frac{4}{3}{(9000+20+36.8)}^{3}- \pi\frac{4}{3}\left( 9000+20 \right)^{3} (20)$

$$P_{theo of MGS}=23-23\frac{{{PV}_{Me}+ V}_{Si in Macro \left( MGS \right)}{+ V}_{C in Macropores}}{{PV}_{G}} (21)$$

All results of this session were used for the finite element model. (details of each term in above equations can be seen in and Supplementary Fig. 2, and Table 1)

**Supplementary Note 4**. **Experimental protocol for finding out maximized volumetric capacity via optimum values of carbon fillings**

To investigate the maximized volumetric capacity via optimum values of carbon fillings, we first identified the ethylene flow time for the carbon filling in MG through controlling the mesopore size distribution. Several-typed MGSs synthesized with different ethylene flow time for 1.5 h (1.5h-MGS), 3 h (3h-MGS), 4.5 h (4.5h-MGS) and 6 h (6h-MGS) were compared as shown in Supplementary Fig. 11, when all samples exhibit the similar gravimetric capacities. Among the various MGSs, 3h-MGS demonstrates the best cycle retention and lowest electrode swelling ratio during full-cell cycling. On this account, we confirmed the ethylene flow time for 3 h is the optimal condition for the maximized volumetric capacity as presented in the manuscript (MGS).

The reason why the excessive carbon filling samples of 4.5h-MGS and 6h-MGS demonstrate capacity degradation is the poreless (poreless means that the samples scarcely contain pores) characteristics. Such phenomenon corresponds with the case of the aGS which scarcely contain pores (Supplementary Fig. 7). To be specific, when the Si is coating on the graphite without pores, it leads to the thick silicon layer with the thickness of ~90 nm and coated graphite does not offer enough space for accommodating the Si volume expansion. The comparison of GS, MGS and aGS reveals that the Si thickness becomes thicker as the pore volumes are decreasing (Supplementary Fig. 16).

On the contrary, the 1.5h-MGS still contains lots of mesopore because of its insufficient ethylene flow time, leading to the generation of Si filling during Si coating process, which results in the poor cycling stability and high electrode swelling ratio (Supplementary Fig. 12). As a result, now that minimum and maximum amount of carbon blocking could give rise to the low volumetric capacity considering the cycling retention and electrode swelling ratio, we finally conclude that 3h-MGS is the best demonstrating the maximized volumetric capacity.
