# Supplementary material for: Towards maximized volumetric capacity via pore-coordinated design for large-volume-change lithium-ion battery anodes
Source: Nat Commun. 2019 Jan 29;10:475. doi: 10.1038/s41467-018-08233-3 (PMC6351620; doi:10.1038/s41467-018-08233-3)
Supplement: Supplementary file 3 — Description of Additional Supplementary Files [file 41467_2018_8233_MOESM3_ESM.pdf]

Description of Additional Supplementary Files

Supplementary Movie 1 :
Supplementary Movie 1 shows diffusion-induced hoop stress of Si-layer-coated graphite (GS) during lithiation.

Supplementary Movie 2 :
Supplementary Movie 2 shows diffusion-induced hoop stress of macropore-coordinated graphite-Si (MGS) during lithiation.
